# Lentil and Kale: Complementary Nutrient-Rich Whole Food Sources to Combat Micronutrient and Calorie Malnutrition

**DOI:** 10.3390/nu7115471

**Published:** 2015-11-11

**Authors:** Megan Migliozzi, Dil Thavarajah, Pushparajah Thavarajah, Powell Smith

**Affiliations:** 1 Department of Agricultural & Environmental Sciences, 270 Poole Agricultural Center, Clemson University, Clemson, SC 29634, USA; megan.migliozzi@gmail.com (M.M.); rajah.thava@gmail.com (P.T.); JPSMTH@clemson.edu (P.S.); 2 International College Beijing, China Agricultural University, 17 Qinghua East Road, Beijing 10083, China; 3 CUCES-Lexington County, 605 W. Main St. Ste. 109, Lexington, SC 29072, USA

**Keywords:** lentils, kale, biofortification, micronutrient malnutrition, obesity, prebiotic carbohydrates

## Abstract

Lentil (*Lens culinaris* Medik.) is a nutritious food and a staple for millions of people. Not only are lentils a good source of energy, they also contain a range of micronutrients and prebiotic carbohydrates. Kale (*Brassica oleracea* v. acephala) has been considered as a health food, but its full range of benefits and composition has not been extensively studied. Recent studies suggest that foods are enrich in prebiotic carbohydrates and dietary fiber that can potentially reduce risks of non-communicable diseases, including obesity, cancer, heart disease, and diabetes. Lentil and kale added to a cereal-based diet would enhance intakes of essential minerals and vitamins to combat micronutrient malnutrition. This review provides an overview of lentil and kale as a complementary nutrient-rich whole food source to combat global malnutrition and calorie issues. In addition, prebiotic carbohydrate profiles and the genetic potential of these crops for further micronutrient enrichment are briefly discussed with respect to developing sustainable and nutritious food systems.

## 1. Introduction

More than 800 million people, or >10% of the world’s population, are suffering from hunger; in certain areas, such as Western Africa and South East Asia, the proportion is >20% [1]. In contrast, Western populations are characterized by significant increases in obesity due to the consumption of calorie-dense foods. This is especially true in the United States, where the 2011–2012 National Health and Nutrition Examination Survey found 35% of adults and 17% of children and adolescents are obese [2]. According to the Global Health Observatory, obesity is linked to 2.8 million deaths per year worldwide [3]. Unbalanced nutrition and obesity are linked to chronic diseases including cardiovascular disease, type 2 diabetes, osteoarthritis, and several cancers [4,5,6]. The urgency created by these and similar life-threatening diseases has made healthy eating a priority for both developing and developed countries. 

Today’s world populations consume inadequate amounts of vegetables, especially nutritionally robust crops, such as leafy greens, but this manifests in different problems. In developing nations, the biggest problem is micronutrient malnutrition; in Western nations, the primary challenge is obesity but micronutrient malnutrition is also present [7]. Two crops that could be paired to address both obesity and micronutrient malnutrition problems seen throughout the world are lentils (*Lens culinaris* Medik.) and kale (*Brassica oleracea* v. acephala). Lentils provide a variety of essential nutrients to a person’s diet, containing high levels of protein (20%–30%), minerals (2%–5%), vitamins (folates), and prebiotic carbohydrates [8,9,10]. Because of their numerous health benefits, high yield, and nitrogen benefit in food systems, lentils are a useful candidate for micronutrient biofortification efforts; however, biofortification research efforts are currently limited to selected crops [10]. Although research has been done on lentils, as well as other pulses, the continued improvement of this crop could be very influential not only for micronutrient malnutrition but also for obesity reduction [10]. On the other hand, kale can be consumed either raw or cooked and is one of the oldest cabbage like plants originating from the eastern Mediterranean region. Kale has received recent attention from health and nutrition sectors due to its nutrient profile, despite the information on which it is based on old varieties, based on a very small sample size, and missing several nutrients kale is likely to contain in considerable amounts of minerals, folates, carotenoids, and prebiotic carbohydrates [11]. With that caveat, kale places high on the list of healthiest foods or also known as a supper food. Kale ranked 15th in a Centers for Disease Control study ranked 47 “powerhouse” fruits and vegetables (a serving providing ≥10% of 17 essential nutrients; [12] that are strongly associated with reducing the risk of heart disease and other non-communicable diseases [12]. Thus, the objective of this review paper is to review the potential of lentils and kale as nutrient-dense whole foods to decrease malnutrition, rates of obesity, and chronic disease-related mortality and make suggestions for future work in this regard.

## 2. Global Micronutrient Malnutrition

**Figure 1:**
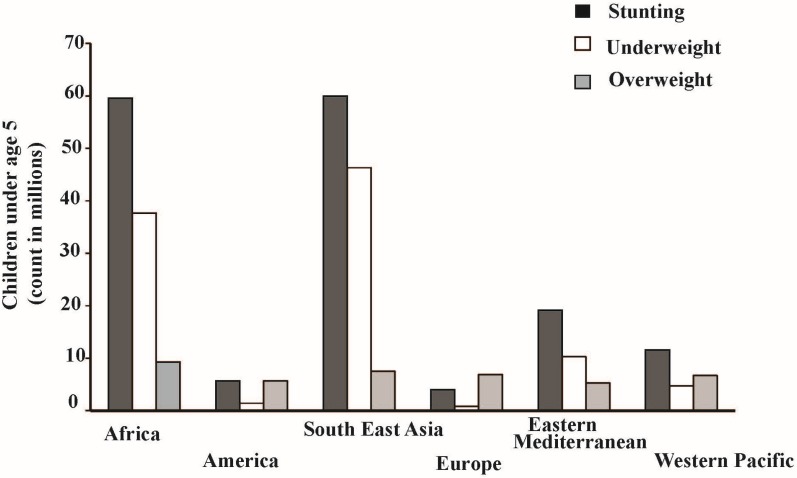
Prevalence of stunting, underweight, and overweight among children under age of 5 (Data adopted from [14]; WHO, 2015).

Micronutrient malnutrition is a global issue. To address hunger in developing nations, past efforts have provided marketable, improved high-yielding cereal crops which are low in essential micronutrients. Indeed, the food produced per capita in 2011 surpassed 2800 kilocalories per day [13]. This is more than enough food to satisfy the caloric needs of the world on a per capita basis. Unfortunately, distribution of food is unequal, leaving billions of people without enough calories to meet daily requirements. Moreover, nutritional health, particularly with respect to micronutrients, has often been overlooked. For example, the introduction of improved high-yield cereal crops that replaced cultivation of more diverse crops resulted in a 20% decrease in vegetable consumption and 40% decrease in fruit consumption in Bangladesh between 1983 and 1995 [5]. Removal of these traditional micronutrient source from daily diets can have wide-ranging impacts. Figure 1 indicates how young children from Africa and Southeast Asia are severely affected by stunting and underweight as a result of micronutrient malnutrition [14]. Approximately 60% of young children under the age of five in Africa are stunted and 35% are underweight (Figure 1). Similar trends of stunting and underweight are observed in South East Asia, however underweight is more prevalent in South East Asia compared to Africa (Figure 1). This issue was recognized in the United Nations Millennium Development Goals, which included a call to reduce the mortality of women and children in Asia and Africa by two-thirds by 2015 using biofortification (breeding crops of increased micronutrient concentration and bioavailability) to improve their micronutrient status [15]. Unlike supplementation and fortification, which add ongoing costs to consumers, biofortification offers the opportunity to change crop nutritional value within the production system and in ways that have little or no impact on consumer cost. For this reason, biofortification is seen as having great potential as a sustainable, food-based solution to global micronutrient malnutrition [16]. HarvestPlus, a non-profit organization introduced biofortified orange-fleshed sweet potato (*Ipomoea batatas* L.) into Africa and Southeast Asia to provide daily β-carotene requirements for malnourished populations [17]. Other crops introduced in a similar fashion include the African eggplant (*Solanum* sp.) and lentils [18,19]. 

Micronutrient malnutrition, also known as hidden hunger, affects more than two billion people globally. The most vulnerable populations are women and preschool children in south Asia, Africa, and Latin America. Estimates indicate that over 60% of the world’s seven billion people are iron (Fe) deficient, over 30% are zinc (Zn) deficient, 30% are iodine (I) deficient, and more than 15% are selenium (Se) deficient. Approximately three million children around the world develop xerophthalmia (damage to the cornea of the eye) and every year more than half a million of children lose eyesight as a result of vitamin A (β-carotene) deficiency. Other micronutrient deficiencies, mainly calcium (Ca), magnesium (Mg), copper (Cu), and vitamin E, are also prevalent in both developed and developing countries [20]. Hidden hunger is also a national concern in the United States, as estimates indicate over 6%–10% of Americans are Fe deficient, over 12% are Zn deficient, 44% are β-carotene deficient, and more than 93% are vitamin E deficient [14]. These deficiencies have significant health effects on unborn children as maternal nutrition has a direct impact on development of the “child *in utero*” or “prenatal development”. In addition, micronutrient malnutrition and obesity also often occur together as a result of nutrient imbalance. 

Initiatives have been established to fight global micronutrient malnutrition. Millennium Development Goals were set in 1990 by the United Nations to reduce worldwide hunger by half by 2015. Undernourishment dropped from 24% in 1990 to 14% in 2013. With recent declines in hunger being much slower than in past years [21], the next revitalization needs to address not only the food supply but also the food quality. Global nutrition targets have been set for 2025 by the World Health Assembly to reduce the percentage of stunted children under the age of 5 and the percent of females who are anemic or Fe-deficient [21]. Therefore, research on food nutrition and the promotion of healthy diets, as well as nutritional breeding of micronutrient-rich crops, will be needed as part of the solution to end micronutrient malnutrition.

## 3. Obesity and Micronutrient Malnutrition 

Obesity is a global issue, as more than 39% of adults are overweight and approximately 13% are obese [22]. However, children from America and Europe are most affected by overweight when compared to global obesity data on a percentage basis. The 2010–2011 National Health and Nutrition Examination Survey reported 35.5% of Americans as obese; 16.9% of children and adolescents were classified as obese [2]. Obesity and overweight status is determined by body mass index (BMI). A BMI above 25 kg/m^2^ characterizes overweight while anything above 30 kg/m^2^ is considered obese. Chronic, non-communicable diseases associated with high BMIs result in an estimated 36 million deaths each year, claiming more lives than all other causes of deaths combined [22]. Non-communicable diseases including diabetes and coronary heart disease are closely linked to obesity related nutritional and metabolic imbalances. Obesity prevalence is higher in countries where economic and nutritional transition occurs. For example, Western countries are already facing obesity as a major health issue and urban areas of developing countries are experiencing obesity as an emerging health issue. Eating habits worldwide have shifted away from legumes, whole grains, and vegetables to high-carb grains, refined sugars, and animal-based products [23]. This nutritional transition, as well as lower costs of highly processed foods and sugary drinks, may be the possible causes for increased obesity, related diseases, and mortality. 

Obesity increases the risk of health conditions including hypertension, adverse lipid concentrations, type 2 diabetes, stroke, gallbladder disease, osteoarthritis, sleep apnea, and several cancers (endometrial, breast, prostate, and colon) [2,4]. In addition, higher body weights are associated with a decreased work force, reduced mobility, social stigmatization, and discrimination [4]. To this end, fighting the American obesity epidemic has been a main focus of many organizations in recent years. The Healthy Weight Commitment Foundation has implemented regulations to remove 6.4 trillion calories from the market through production regulation and policies [24]. Numerous other steps have been taken to implement healthy school lunch programs to reduce the intake of refined sugars and promote physical activity. In countries where the effects of obesity are high, government regulations aim to deter the population from consuming calorie dense foods. For example, the Obesity Prevention Strategy of Mexico was created in 2010 to aid in the removal of beverages high in sugar and fat content from schools [23]. Choices International Foundation, created in 2006, is an international program that promotes food products through scientific analysis and encourages front-of-package nutritional labeling. This has given consumers greater knowledge and an opportunity to choose healthier foods. In 2004, the World Health Assembly adopted the Global Strategy on diet, physical activity, and health to prevent non-communicable diseases, *i.e.*, obesity, by creating policies and programs promoting healthy eating and exercising habits [25]. 

Several approaches can be applied to the management of overweight and obesity. Dietary therapies (low caloric diets or lower fat diets), changes in physical activity and/or social behaviors, surgical procedures, and combinations of these options are available. Malhotra and others claim that poor diet is the leading cause of obesity in the Western world [26] and therefore solutions will necessarily have a focus on diet [4]. With a loss of agricultural ties and an ever-changing variety of food products over the last several decades, Western diets have shifted to calorie-dense foods high in refined sugars, carbohydrates, and fats [23]. This shift away from fruits, vegetables, and legumes has had dramatic impact on population health. Additionally seen is a trend that links lower economic groups within these populations to higher rates of obesity, with the most likely being that “calorie-dense foods” are cheap, fast, and easy. Big industrial food operations produce food products that can be mass produced at low cost and are accessible to all [23]. However, these food-like products are made from only a few main ingredients, are highly processed, and are devoid of desired nutritional components. Moreover, few Americans consume the recommended amount of fruits and vegetables and the Dietary Guidelines Advisory Committees of both 2010 and 2015 have called for increased intakes of these underconsumed foods [27]. Therefore, not only is the shift in Western diets causing obesity due to overconsumption of sugars, starches, and fats, it is causing micronutrient deficiencies as well. 

## 4. Food Systems Approach

In order to combat both obesity and micronutrient malnutrition, the concentration of food nutrients and their true bioavailability to humans must be highlighted. Welch and Graham (2005) extensively reviewed the potential for micronutrient promotion through staple food crops including legumes, roots, and vegetables [28]. Biofortification enhances the nutritional quality of crops, such as pulses and vegetables, using breeding techniques and biotechnology. The biofortification approach allows the most nutrient-rich varieties to be cultivated without a decrease in their biological yield [15,28]. By improving the nutritional composition and human bioavailability of food matrices through breeding and processing, these crops can be promoted for inclusion in a healthy diet and lessen the risk of disease development for millions of people in developing countries. 

Welch suggests a new approach to agriculture, considering “food systems” more greatly and focusing less on yield and quantity in production [29]. This approach gives more attention and consideration to the nutrition provided by the crops cultivated. Since the “Green Revolution”, which was initiated to combat hunger, staple crops such as sugar cane, wheat, corn, and rice have been the main focus of modern agriculture. Diversity and variety was compromised, with severe declines in fruit, vegetable, and legume production and consumption [29]. This loss of diversity in the diet is one of the greatest factors contributing to the calorie and micronutrient malnutrition seen globally [5]. Studies have emphasized the importance of legumes in agricultural food systems [10,28]. Legume production and consumption have the potential to improve not only the health of the consumer but also agriculture by returning available nitrogen to the soil through nitrogen fixation. Leafy green vegetables also have the potential to increase the nutritional value of a diet by providing many critical micronutrients and minerals. More research needs to be done on these and similar food matrices to increase the likelihood that a second agricultural “revolution” will be successful and sustainable for the future. 

## 5. Nutrient Density, Bioavailability, and Prebiotic Carbohydrates

Nutrient density and bioavailability of micronutrients are equally important for achieving optimal nutritional status of a diet. Nutrient density is a measurement of nutrients in a food per calorie or unit weight. Bioavailability is the proportion of an ingested nutrient in human diet that is absorbed and utilized for essential metabolic functions in the human body. Therefore, bioavailability is an important factor to consider when the aim is to prevent malnutrition and obesity [28,29,30]. Bioavailability of a micronutrient is governed by many factors: host, digestive environments, and the presence of mineral absorption promoters and inhibitors in a food. For example, mineral absorption promoters, such as ascorbic acid, carotenoids, prebiotic carbohydrates, fibers, sulfur containing amino acids, and meat factors, increase iron absorption in the human digestive system; phytic acid and polyphenols in plant-based food are the major inhibitory factors of Fe and Zn bioavailability [29]. 

Prebiotics are an important, and yet often overlooked, food component. Escaping digestion, prebiotics are a class of complex carbohydrates that cannot be broken down by the human enzymatic digestion, are fermented by bacteria in the microbiome, and selectively increase the population of certain species of helpful bacteria [31]. Recent discoveries suggest the intestinal microbiome and a prebiotic-rich, low-caloric diet can play important roles in combating obesity and related diseases [32,33]. Additionally, consumption of non-digestible, fermentable carbohydrates (or prebiotics) may stimulate the growth and activity of hind gut bacteria [34] by producing short-chain fatty acids that provide an energy source for colonocytes, strengthen the gut mucosal barrier, and suppress colonization of pathogens [35]. As a result, products enriched with prebiotic carbohydrates are becoming more popular health-promoting foods in the world. Naturally occurring prebiotic carbohydrates are categorized into two major groups: dietary fiber and sugar alcohols [36]. Dietary fiber is comprised of starch polysaccharides including resistant starch (RS) and non-starch polysaccharides, such as raffinose-family oligosaccharides (RFO) and fructooligosaccharides (FOS) [36]. Based on this definition, so far very few carbohydrates have actually been classified as prebiotics. For example, many starches which show prebiotic characters, including glucooligosaccharides, polydextrose, xylooligosaccharides and others, remain unclassified as such without evidence for their ability to increase populations of specific bacteria *in vivo* [36]. 

Roberfroid (2007) indicated that the only two oligosaccharides shown to fulfill all requirements for prebiotic classification are inulin and *trans-*galactooligosaccaride (TOS) [36]. Both have been extensively researched, with evidence from *in vitro* and *in vivo* experimental trials supporting their classification as prebiotics. For example, TOS escapes digestion and effectively increases the population of bifidobacteria and lactobacilli while decreasing the number of enterobacteria [36]. Many different food sources may contain potential prebiotic carbohydrates. However, because the presence of true prebiotics in food is minimal, foods that contain significant amounts of them are very important. 

Prebiotics are key to human gut health. The human intestine holds a complex and diverse microbial community referred as “gut microflora” or “microbiota”. Human gut microbiota are comprised of approximately 10^14^ bacteria and archaea with nearly 1100 prevalent species. Gut microflora contain 150-fold more genes than the human genome [37,38]. In addition, human gut microbiota predominantly belong to four bacterial phyla: Gram-negative *Bacteroidetes* and *Proteobacteria* and Gram-positive *Actinobacteria* and *Firmicutes* [37,38]. Ley *et al*. (2005) provided the initial evidence of gut microflora associated with obesity using the leptin-deficient *ob/ob* mouse model [39]. The gut microflora of *ob/ob* mice had significantly reduced numbers of *Bacteroidetes* and a proportional increase in *Firmicutes* compared to lean *ob/+*, wild-type siblings, and their *ob/+* mothers when all fed the same diet. Feeding a high-fat diet to genetically wild-type rodents also demonstrated the same microbial changes. The relative proportion of *Bacteroidetes* is decreased in obese individuals compared to lean individuals; however, this relative proportion rebounds with weight loss on a prebiotic-rich, low-caloric diet [40]. Furthermore, consumption of non-digestible, fermentable carbohydrates (or prebiotics) may stimulate the activity of hind gut bacteria by producing short-chain fatty acids that provide an energy source for colonocytes, strengthen the gut mucosal barrier, and suppress colonization of pathogens [34,35]. 

In mammals, diet and phylogeny influence the bacterial diversity of gut microbiota. A complex living system, the microbiome aids in many processes required for life. The bacteria line the inner walls of the intestine, mainly in the large intestine. There, they metabolize food byproducts that have escaped digestion in the upper digestive system. Prebiotics that cannot be digested and absorbed by human physiological processing alone must be fermented by these bacteria. Profound changes in diet and lifestyle conditions were observed with the introduction of agriculture and animal husbandry ≈10,000 years ago. Western diets, common for those living in developed regions of the world. *i.e.*, Northern Europe and the United States, are associated with certain types of gut microflora and linked with obesity trends [41]. Children from Africa, who ate a diet high in fiber and plants and low in fat, showed higher levels of *Bacteroidetes*; children from Europe, who ate a Western diet high in animal fats and sugar and low in fiber, showed higher levels of *Firmicutes*. Overall, this study clearly revealed the differences in bacterial composition of the gut depending on diet, strengthening the argument that improving gut health through modification of diet rich in prebiotic carbohydrates is possible. 

These possible host health benefits are mainly due to fermentation by colonic bacteria of dietary prebiotic carbohydrates, which produce short chain fatty acids (SCFAs) such as acetic, n-butyric, and propionic acid. Acetate is used as a substrate for liver cholesterol and fatty acid synthesis, and increases colonic blood flow and oxygen uptake [42]. Butyrate acid acts as an energy source for colonocytes and prevents tumor growth and differentiation of colon epithelial cells [43]. Propionic acid is a major metabolite that lowers lipogenesis, serum cholesterol levels, satiety, and risk of colon cancer [35]. In relation to obesity, SCFA concentrations are shown to differ between groups of lean subjects and overweight/obese subjects; SCFA concentration and the ratio of *Firmicutes* to *Bacteroidetes* in the gut were both generally elevated in the obese group [44]. There is speculation that elevated SCFA concentrations result from either increased SCFA production or decreased absorption in the gut of obese individuals and is directly related to obesity. 

Each variety of bacteria in the gut plays some role in the human body, either positive or negative. Different bacterial varieties are able to metabolize different compounds, breaking down and utilizing specific food components. Moreover, each bacteria is important due to its unique role in the human gut. However, some bacteria are more beneficial to human health and their growth could be encouraged through specific choices in diet. Through the processes carried out by the microflora, nutrients that may not have been readily available in food in the form consumed may become accessible. By metabolizing certain food components, the bacteria release nutrients in a more bioavailable form to human absorption, increasing nutrient absorption [31]. This is extremely important when a food matrix contains high levels of a crucial micronutrient that may be in a chemical state that the body cannot absorb directly. It is for this reason, and others, that the health of the human gut and its microbial community is so crucial to overall health. With a well-maintained microbiome, the nutritional vigor of a healthy diet can be improved further. 

A survey of American diets estimated that consumption of prebiotic carbohydrates ranges from 1 g to 10 g per day per person [45]. The Institute of Medicine set the Acceptable Macronutrient Distribution Range for carbohydrates at 45%–65% of total energy intake. The Adequate Intake of total fiber is 38 g for men and 25 g for women. Although many prebiotic carbohydrates are categorized as fiber, no specific official recommendations have been made regarding their consumption [46]. However, the major source of dietary prebiotic carbohydrates in most Western diets is wheat, which, as mentioned above, does not provide equivalent amounts of prebiotics compared to traditional legumes, fruits, and vegetables. Therefore, traditional whole food approaches are gaining wide acceptance but little is known about the prebiotic profile of many whole foods, including pulses such as lentil and leafy green brassica vegetables such as kale.

## 6. Lentils

Today, approximately five million tons of lentil are produced around the world [13]. The major lentil producing countries are Canada, India, Turkey, Australia, and the USA. Lentils have been a key source of protein (20%–30%) for a very long time in many different cultures, especially in Southeast Asia, Africa, and Mediterranean regions. Lentils are an excellent source of essential macro- and micronutrients, including prebiotic carbohydrates, folates, and minerals (Table 1). 

**Table 1:** Nutritional composition analysis of lentils grown in the USA and Canada [9,11,46].

Nutrient component (units)	Concentration
Moisture (%)	1–12
Protein (%)	20–29
Ash (%)	1.8–3.3
Total lipid (fat) (%)	1–2
Carbohydrate, by difference (%)	60
Total starch (%)	40–70
Total prebiotic carbohydrates (g/100g)	12.3–14.1
Calcium, Ca (mg/kg)	460–496
Iron, Fe (mg/kg)	73–90
Potassium, K (mg/kg)	6954–7761
Zinc, Zn (mg/kg)	44–54
Selenium ((µg/kg)	425–673
Ascorbic acid (mg/kg)	61.2–84.3
Folate, Dietory Folate Equalents (µg/g)	2.2.–2.9
Phytic acid (mg/g)	2.4–4.4

Lentils are typically rich in micronutrients and have the potential to provide adequate dietary amounts, especially for iron (Fe), zinc (Zn), and selenium (Se); a 50 g serving provides 3.7–4.5 mg Fe, 2.2–2.7 mg Zn, and 22–34 µg Se [18]. Lentils are also a good source of vitamin A, thiamin, folate, and β-carotene and also contain considerable amounts of riboflavin, niacin, pantothenic acid, pyridoxine, vitamin K, and vitamin E [8]. The limited availability of these vitamins in common food sources makes their presence in lentil highly valuable. In addition to vitamins and minerals, lentils contain other phytonutrients including flavonoids, tannins, phytic acid, phytosterols, and many others. The health benefits of phytonutrients vary and include but are not limited to anti-inflammatory, antioxidant, and anticancer effects [47]. Unlike other grains, lentils are very low in phytic acid (2.5–4.4 mg·g^−1^); this is advantageous because phytic acid binds Fe and Zn and thus makes them poorly bioavailable [18].

Several approaches have been used to determine Fe bioavailability in plant foods, including *in vitro* digestion/Caco-2 cell culture, animal (rodent, poultry, and pig) models, and human studies [48]. Currently, the *in vitro* digestion/Caco-2 cell culture method has been used to estimate Fe bioavailability for food crops including rice, soybean (*Glycine max* L.), common bean (*Phaseolus vulgaris* L.) and lentils [49]. Lentil Fe bioavailability using this *in vitro* digestion/Caco-2 cell culture model show very high levels of ferritin formation (22.0–7.2 ng/mg of protein; mean 13.1 ng/mg). Other staple food crops such as wheat (5.29 ng/mg of protein), finger miller (6.98 ng/mg of protein), and red kidney beans (3.81 ng/mg of protein) are comparatively low in Fe bioavailability [18,49]. To investigate lentil as a source of Fe to anemic children, a clinical nutrition study in Sri Lanka was initiated. A pilot study with 33 mild anemic children (hemoglobin levels = 11 ± 0.8 g/dL) shows that the group fed 50 g of red lentils (Crop Development Center (CDC) Redberry) per day for two months had significantly improved their Fe status (Table 2; [50]). These preliminary results clearly show consumption of red lentils (CDC Redberry) can improve the Fe nutritional status of anemic children in Sri Lanka, and thus have potential for other populations as well. 

**Table 2:** Percent improvement of mild anemic children (*n* = 33) in Sri Lanka after 60 days of red lentil feeding trial [50].

Indicator	0 days	60 days	% Improvement
Hemoglobin (g/dL)	11.1	11.8	6.3
Serum Fe (µg/dL)	51.5	89.8	74.4
Total iron binding capacity (µg/dL)	405.3	377.6	−6.8
Trans ferritin saturation (%)	12.8	24.3	89.8
Serum ferritin (ng/mL)	29.5	41.2	39.7

**Figure 2:**
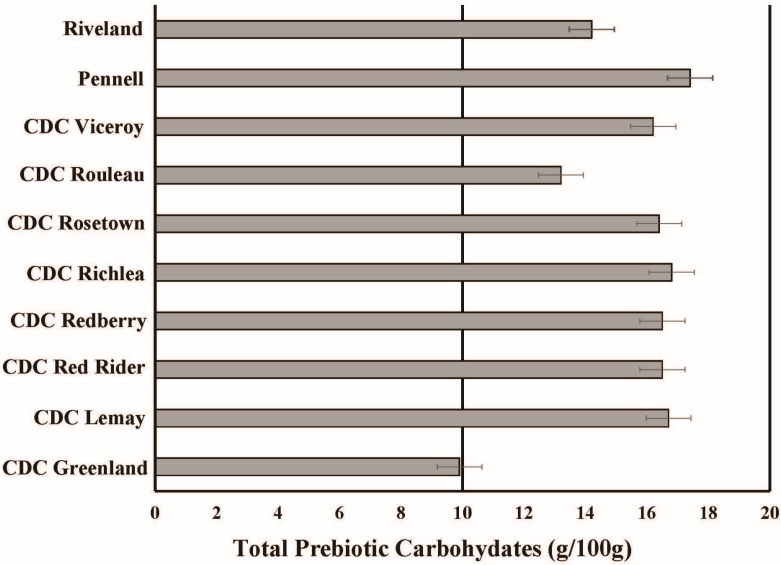
Concentrations of total prebiotic carbohydrates in a 100 g serving of different lentil cultivars grown the USA. Original data adopted from Johnson *et al*. (2013) [46]. Total prebiotics are the sum of sugar alcohols, raffinose oligosaccharides, fructooligosaccharides, and resistant starch. Recommendation for daily total prebiotic intake reported by Douglas and Sanders (2008) is 10–20 g per day [51].

In addition to minerals, lentils are rich in prebiotic carbohydrates including resistant starch and other non-digestible starches, providing over 13 g per 100 g serving [46]. Resistant starch is a complex carbohydrate that is not digested in the gut and instead acts as a prebiotic, being fermented by bacteria. Mean concentrations of resistant and total starch for lentils are 7.5 and 47 g 100 g^−1^, respectively [46]. Across 10 varieties grown in the USA, average resistant starch ranged from 6.0 g 100 g^−1^ in CDC Greenland to 8.9 g 100 g^−1^ in Pennell; total starch ranged from 45 to 48 g 100 g^−1^. In addition, all commercial lentil market classes evaluated were relatively high and uniform with respect to total prebiotic carbohydrate concentrations (Figure 2; [46]). Therefore, lentils offer new opportunities as a whole food to promote gastrointestinal health to reduce obesity.

## 7. Leafy Green Brassica: Kale 

Brassica vegetables, such as kale and collard greens (*Brassica olceracea* var. Acephala), are very important to many agricultural systems around the world. The estimated annual value of brassica vegetables in the USA is approximately $1 billion. Broccoli, cabbage, cauliflower, and Brussels sprouts are the most valuable brassica crops; however, no production statistics are currently available for leafy green brassicas in the USA. Kale, traditionally a less recognized crop, is becoming a significant specialty crop in the Southern US as a result of its suitability to southern fall and winter growing conditions. Kale is a traditional leafy green brassica used as a garnish on plates and salad bars but is gaining popularity as a primary ingredient in either raw or cooked form. Georgia, North Carolina, and South Carolina have emerged over the last five years as the leading kale producing states. From some 500 acres producing only 15% of US kale in 2001, the Carolinas have emerged as kale leaders, currently producing more than two-thirds of America’s annual kale output. Most of the kale produced in this region is sold in a variety of fresh and processed forms that are nationally distributed. Carolina kale is marketed by market class (curly, dinosaur, red Russian, ornamental), stage of maturity, and packed fresh ready-to-eat/cook. Kale is also a popular leafy green vegetable in northern regions of Asia, and Europe. Between 1993 and 2013, about 72.3% of the world’s brassica vegetables were produced in Asia. The production rate of brassica vegetables for human consumption was 270 million metric tons as of 2012 but is small in comparison with sugarcane, soybean, and maize, for which about one billion tons were produced [13]. Worldwide, the production of meats, sugar, and cereal grains has greatly overshadowed the production of more diverse and healthful vegetable crops, including nutritionally crucial cultivars such as brassica greens.

These green vegetable crops can provide much needed nutrients to consumers and are often proclaimed as health foods because of their nutrient-rich composition. Brassica vegetables contain numerous micronutrients, such as antioxidants, carotenoids, glucosinolates, polyphenols, vitamins, and minerals important to human health [52]. Available data indicate that kale is rich in several vitamins (A, K, C, and probably folate), essential minerals (potassium, calcium, magnesium), and dietary fiber. It is likely that kale can also provide other nutrients including carotenoids, folate, and prebiotic carbohydrates, although these have not been characterized [53]. Brassica greens are also known to contain phytochemicals such as folic acid, ascorbic acid, riboflavin, and carotenes [54]. Flavonoids act in the body as antioxidants and capture free radicals. This means that they may have a lessening effect on the likelihood of developing chronic diseases such as cancer. Other phytochemicals found in vegetables such as brassicas are categorized as anti-nutrients. These chemical compounds are known to disrupt many physiological pathways and lessen the absorption of beneficial nutrients. Included in this group are oxalates, phytate, and tannins. 

Despite its gaining popularity, kale remains an understudied vegetable. In the Science Direct database, 636 publications are returned using “kale” as a keyword but only four relevant studies when using “kale and nutrition”. These include three studies from Brazil, Turkey, and Pakistan [55,56], and one study from the USA [57]. No studies of kale nutritional quality appear to have been carried out in the last few decades. However, significant genomic research on broccoli has been carried out at the University of Wisconsin, University of California, University of Georgia, and Cornell University. The specific focus of these research studies was on genomics and gene identification to determine mutation profiles of brassica [58]. 

## 8. Future Implications

Both lentils and kale have the potential to be extremely beneficial staple crops worldwide. They are nutrient-dense food matrices, containing a wide variety of macro- and micronutrients. Many studies with pulse crops, and lentils in particular, confirm their nutritional value to human diets. Fewer such data are available for kale, but recent attention focused on this leafy brassica indicate its potential to be one of the healthiest foods in terms of nutrient value. It is speculated that kale, like pulses, may also contain specific micronutrients, such as dietary fiber, which can act as prebiotics in the human gut and increase nutrient absorption overall. By studying the nutritional composition of lentil and kale, increasing consumption of micronutrient-rich varieties, and improving bioavailability of those micronutrients, the health of the consumer can be greatly improved. These crops have the potential to reduce calorie malnutrition, micronutrient deficiencies, and chronic disease around the globe if properly researched, biofortified through biotechnology and breeding programs, and promoted. 

An additional consideration is preparation. Processing and cooking impact the nutritional profiles of these food crops and must be considered with respect to nutrient bioavailabilities. For example, resistant starch levels in lentils change significantly after processing, cooking, and cooling [59]. Thus, certain processing techniques may allow consumers to further increase their nutritional value [60] while other techniques may affect micronutrient concentration or composition in a negative way. By discovering the effects of different processing procedures, it will be possible to select the techniques that improve the nutritional quality of these foods. Understanding these effects for different foods can help consumers make conscious choices with respect to both preparation and consumption to preserve nutritional quality. 

In a food systems approach, brassicas are the perfect complement to pulse crops. Pulse crops fix their own nitrogen through a symbiotic relationship with *Rhizobium* bacteria. This characteristic of legumes is what makes them advantageous as a cover crop or rotation crop. Because pulses return available nitrogen to the soil, they can improve the yield and nutritional quality of following (or subsequent) crops. Thus, subsistence farmers would benefit economically from growing pulses and brassica vegetables together through improved soil quality and perhaps even yield; their own diet would also benefit [28]. Not only lentils, other cool season pulses including field pea and chickpea are also a good source of micronutrients and prebiotic carbohydrates. For brassica vegetables, broccoli, cauliflower, Brussels sprout, and collards are also good source of micronutrients. Therefore, balancing vegetarian diet with legumes and brassica vegetables can greatly improve the nutritional quality. 

In a world where so many people are struggling to eat, considering both quantity and quality of food is important. Typical diet in low income countries is mainly cereal based, however lentils are also the most affordable and culturally acceptable protein source for low income families in South Asia. Our field data from Nepal, India, Bangladesh, and Sri Lanka show individual average daily lentil consumption ranges from 15 to 30 g (personal communication with Dr. Shiv Kumar, ICARDA [61]). Thus, in countries where millions of people are suffering from hidden hunger, increasing the micronutrient content of staple foods could significantly address micronutrient malnutrition. Thus, we believe that lentils containing nutritionally significant amounts of bioavailable minerals and vitamins can make important contributions to public health, particularly in South Asia. Research plays a key role in the ability to increase the nutritional value and micronutrient content in foods, such as pulse and brassica crops, to benefit a range of consumers around the world. If introduced into agricultural systems where calorie malnutrition and micronutrient deficiencies are a prevalent issue, these crops could help restore micronutrient levels and therefore improve health in those populations. Promotion of these foods within populations that are struggling to improve their diets and regain good health could bring about significant results. A better understanding of these and many other food matrices will allow global populations to make healthier choices. Diet is becoming a key strategy to prevent disease and improve other health conditions. By changing one’s diet to include certain nutrients, each person can drastically influence their own health [15]. If it is known what food processing effects on nutritional compositions, what the most nutritional varieties are, and which forms of nutrients are the most bioavailable, we can choose to eat food in the way that will be most beneficial to our health. 
